# Cost-Effectiveness Analysis of Camrelizumab Versus Chemotherapy as Second-Line Treatment of Advanced or Metastatic Esophageal Squamous Cell Carcinoma

**DOI:** 10.3389/fphar.2021.732912

**Published:** 2021-11-16

**Authors:** Hongfu Cai, Baohua Xu, Na Li, Bin Zheng, Zhiwei Zheng, Maobai Liu

**Affiliations:** ^1^ Department of Pharmacy, Fujian Medical University Union Hospital, Fuzhou, China; ^2^ College of Pharmacy, Fujian Medical University, Fuzhou, China; ^3^ Department of Pharmacy, Cancer Hospital of Shantou University Medical College, Shantou, China

**Keywords:** cost-effectiveness, esophageal cancer, esophageal squamous cell carcinoma, chemotherapy, camrelizumab

## Abstract

**Background:** This study aimed to analyze the cost effectiveness of camrelizumab in the second-line treatment of advanced or metastatic esophageal squamous cell carcinoma in China.

**Methods:** On the basis of the ESCORT clinical trial, a partitioned survival model was constructed to simulate the patient’s lifetime quality-adjusted life years (QALYs), lifetime costs, and incremental cost-effectiveness ratio (ICER). One-way sensitivity and probability sensitivity analyses were performed to test the stability of the model.

**Results:** Treatment of esophageal squamous cell carcinoma with camrelizumab added 0.36 QALYs and resulted in an incremental cost of $1,439.64 compared with chemotherapy, which had an ICER of $3,999 per QALY gained. The ICER was far lower than the threshold of willingness to pay for one time the GDP per capita in China. Sensitivity analysis revealed that the ICERs were most sensitive to the cost of drugs, but the parameters did not have a major effect on the results of the model.

**Conclusion:** Camrelizumab is likely to be a cost-effective option compared with chemotherapy for patients with advanced or metastatic esophageal squamous cell carcinoma. This informs patient selection and clinical path development.

## Introduction

The world has approximately 572,000 new cases of esophageal cancer and 508,000 deaths every year. The number of new cases of and deaths from esophageal cancer in China ranks sixth and fourth places among all malignant tumors, respectively (Sung et al., 2021). Esophageal cancer could be divided into esophageal squamous cell carcinoma, esophageal adenocarcinoma, and other subtypes. Esophageal squamous cell carcinoma accounts for more than 90% of esophageal cancer (Arnold et al., 2015). The incidence of esophageal squamous cell carcinoma is increasing in some Asian countries. Half of the global esophageal squamous cell carcinoma cases occur in China (Zhang et al., 2012; Sung et al., 2021). Most patients are already at an advanced stage when they are diagnosed with local invasion or distant metastasis. The prognosis of advanced or metastatic esophageal squamous cell carcinoma is poor, and the overall 5-year survival rate is less than 20% (Torre et al., 2016). Therefore, advanced or metastatic esophageal squamous cell carcinoma has gradually become a more difficult problem in the treatment of tumor diseases.

Platinum drugs combined with fluorouracil or Paclitaxel are the standard first-line treatment option for the treatment of esophageal squamous cell carcinoma, but after the progress of first-line treatment, the choice of second-line treatment is limited (Shah, 2015; Kojima et al., 2020). The median survival time after failure of first-line chemotherapy is only 5–10 months (Sun et al., 2021). Thus, finding more cost-effective second-line treatment drugs is very important. Immune checkpoint inhibitors (ICIs) are more effective and has lower incidence of adverse reactions than chemotherapy. They have become the choice of second-line treatment for patients (Kojima et al., 2020). Camrelizumab, a high-affinity, fully humanized, selective IgG4-κ monoclonal antibody against PD-1, has shown activity across a wide range of solid tumors (Fan et al., 2021; Peng et al., 2021; Yang et al., 2021; Zhou et al., 2021). The Chinese guidelines recommend camrelizumab as a second-line treatment for distant metastatic esophageal cancer (CSCO, 2020). The ESCORT study has shown that second-line camrelizumab significantly improved the overall survival (OS) of patients with advanced or metastatic esophageal squamous cell carcinoma compared with chemotherapy, with a manageable safety profile (Huang et al., 2020).

Camrelizumab officially entered China’s National Medical Insurance in March 2021, with a price reduction of 85.2% (Nation Healthcare Security Administration, 2020). After the price reduction, whether camrelizumab may become an cost-effective second-line treatment has become an issue of great interest to medical insurance, doctors, and patients. Therefore, in this study, a cost-effectiveness analysis of camrelizumab was conducted in the second-line treatment of advanced or metastatic esophageal squamous cell carcinoma.

## Materials and Methods

### Model Structure

**FIGURE 1:**
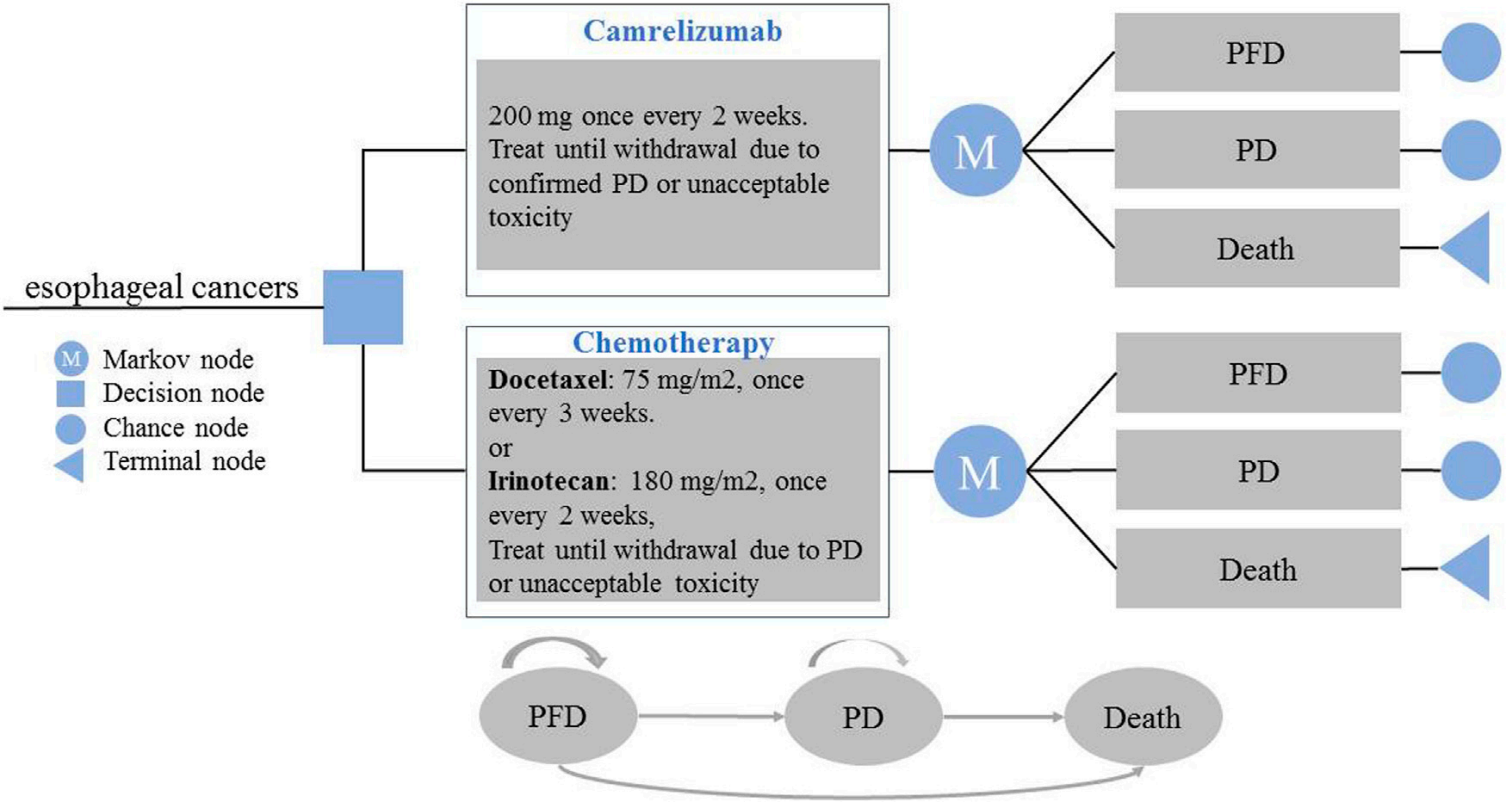
Model Structure of a Decision Tree Combining the Partitioned Survival Model. PFD, progression-free disease; PD, progressive disease.

The target population of the study was patients with advanced or metastatic esophageal squamous cell carcinoma who previously failed to receive first-line chemotherapy. The patients were assigned to receive either camrelizumab or chemotherapy (docetaxel or irinotecan). A partitioned survival model was established to reflect the disease progression. The model included three states: progression-free disease (PFD), progressive disease (PD), and death. The three states are mutually exclusive. All patients were assumed to enter the model in the PFD state, and that they could maintain their designated health state or develop into another health state in each cycle (Figure 1). The relative 5-year survival rate is 8% or less for patients diagnosed with metastatic disease; thus, the time horizon of the model was set to 10 years (ASCO, 2020; Cancer Information Service, 2020). The model period was set to 1 month to facilitate model operation and parameter calculation. The main results of the model output were total cost, incremental cost-effectiveness ratio (ICER), and quality-adjusted life years (QALYs). ICER refers to the additional cost required for each additional QALY. Cost and utility were discounted at a rate of 5% (Liu, 2020). All costs were converted to USD, with an average RMB exchange rate of $1 to 6.8974 Yuan for the full year of 2020 (National Bureau of statistics of China, 2020). In addition, 1–3 times the national per capita GDP in 2020 ($10,503.52) was used as the willingness-to-pay (WTP) threshold (World Health Organization, 2011; Liu, 2020; National Bureau of statistics of China, 2020). The TreeAge Pro 2020 software package was used to build the model and conduct statistical analysis.

### Clinical Data

The clinical efficacy and safety data of the second-line treatment for advanced or metastatic esophageal squamous cell carcinoma were mainly from the ESCORT clinical trial (Huang et al., 2020). The ESCORT study is a randomized, open, multi-center phase III clinical study. It is the world;s first and largest clinical study of camrelizumab in Chinese patients with advanced/metastatic esophageal squamous cell carcinoma who previously failed first-line chemotherapy. A total of 228 patients received camrelizumab monotherapy, and 220 patients received chemotherapy (docetaxel or irinotecan). The eligible patients had histological or cytological diagnosis of esophageal squamous cell carcinoma; advanced, recurrent, or distant metastatic disease; and showed progression on previous first-line chemotherapy. The main exclusion criteria included CNS metastases and a history of anti-PD-1 or anti-PD-L1 therapy (Huang et al., 2020). GetData Graph Digitizer 2.26 (http://www.getdata-graph-digitizer.com) was used to obtain points on the survival rates of the Kaplan–Meier curves. Following Hoyle et al.(Hoyle and Henley, 2011), R software was used to reconstruct the individual data, which were then fitted by exponential, gamma, gompertz, Weibull, loglogistic and lognormal distribution. The best fitting distribution was selected by visual inspection, the lowest value of the Akaike information criterion (AIC), and Bayesian information criterion (Zhang et al., 2020a) (Supplementary Table S1). The log-logistic distribution function was used to simulate the PFS and OS curves of the two schemes. We performed internal and external model validations (Goldstein et al., 2015). The internal validation showed that the PFS and OS curves closely approximated those presented in the clinical trials (Supplementary Figures S1, S2). In the external validation, we compared the survival curves used in this study with those for the same treatment in other published studies. PFS and OS curves were compared with those from ORIENT-2 study (Xu et al., 2020), as shows in Supplementary Figures S3, S4. The survival function of log-logistic distribution at time t was S(t) = 1/(1+λt^γ^) to calculate the scale parameter *λ* and the shape parameter *γ* (Ishak et al., 2013; Diaby et al., 2014). The Weibull distribution as the next best fitting function was also used to project lifetime survival curves in our model. The key clinical inputs are given in Table 1.

**TABLE 1:** Basic parameters input to the model and the ranges of the sensitivity analyses.

Variable	Baseline value	Lower limit	Upper limit	Source
Log-logistic PFS survival model				
Camrelizumab	γ = 2.0011; *λ* = 0.1471	—	—	Huang et al. (2020)
Chemotherapy	γ = 3.1368; *λ* = 0.08394	—	—	Huang et al. (2020)
Weibull PFS survival model				
Camrelizumab	γ = 1.1296; *λ* = 0.1965			Huang et al. (2020)
Chemotherapy	γ = 1.9527; *λ* = 0.1282			Huang et al. (2020)
Log-logistic OS survival model				
Camrelizumab	γ = 1.2879; *λ* = 0.04461	—	—	Huang et al. (2020)
Chemotherapy	γ = 2.1592; *λ* = 0.01946	—	—	Huang et al. (2020)
Weibull OS survival model				
Camrelizumab	γ = 1.3018; *λ* = −0.04380			Huang et al. (2020)
Chemotherapy	γ = 1.4487; *λ* = 0.04345			Huang et al. (2020)
Health utilities				
Progression-free disease	0.741	0.593	0.889	Zhang et al. (2020b)
Progressive disease	0.581	0.465	0.697	Zhang et al. (2020b)
Anemia	−0.074	−0.037	−0.110	Tan et al. (2018)
Decreased neutrophil count	−0.090	−0.059	−0.120	Tan et al. (2018)
Vomiting	−0.048	−0.016	−0.080	Tan et al. (2018)
Drug cost per mg, US $				
Camrelizumab	2.16	1.08	2.16	Nation Healthcare Security Administration. (2020)
Docetaxel	1.77	0.26	14.95	YoaZH. (2020)
Irinotecan	1.64	0.88	4.65	YoaZH. (2020)
Drug administration costs, US $				
Follow-up cost per cycle	7.47	6.52	8.47	Zhang et al. (2020b)
Best supportive care cost per cycle	16.98	4.68	46.77	Zhang et al. (2020b)
SAE management cost, US $				
Anemia	73.68	55.27	92.11	Zhang et al. (2020b)
Decreased neutrophil count	67.56	200.66	55.27	Zhang et al. (2020b)
Vomiting	98.33	63.64	140.46	Guy et al. (2019)
Body surface area, m^2^	1.72	1.50	1.90	Zeng et al. (2013)
Discount rate	0.05	0	0.08	Liu. (2020)

PFS, progression-free survival; OS, overall survival; SAE, severe adverse event.

### Cost and Utility

The study only considered direct medical costs, including drug acquisition, follow-up, best supportive treatment, and severe adverse event (SAE) management costs. In accordance with the ESCORT clinical research and guidelines (CSCO, 2020; Huang et al., 2020), 200 mg carrelizumab was administered intravenously on the first day of every 2 weeks, 75 mg/m^2^ docetaxel was provided on the first day of every 3 weeks, and 180 mg/m^2^ of irinotecan was administered intravenously on the first day every 2 weeks. Treatment was continued until disease progression or unacceptable toxicity. The cost effectiveness of the two scenarios was discussed to avoid the effect of the course of drugs on the results. In the first scenario, camrelizumab was assumed to be used for six (IQR 4–13) courses, docetaxel for three (2–3) courses, and irinotecan for four (2–5) courses in accordance with the results of ESCORT (Huang et al., 2020). A shorter time horizon (3, 5 and 7 years) was also considered in this scenario. In the second scenario, both groups continued treatment until the disease progressed. The proportion of patients receiving specific chemotherapy regimens was not defined in the clinical trials. The model assumed that the patients had equal opportunities to receive docetaxel and irinotecan. The average body surface area of the patients in the model was 1.72 m^2^ (1.5–1.9 m^2^) (Zeng et al., 2013). After the failure of second-line treatment, the best third-line treatment was not clear, and the specific scheme was not shown in the ESCORT study. Therefore, the best support treatment was regarded as the treatment after progression.

The cost of camrelizumab was derived from the negotiated price of China’s national medical insurance (Nation Healthcare Security Administration, 2020). The cost of docetaxel and irinotecan was the median of the bidding price of drugs in different provinces (YaoZH, 2020). Only the SAE of grade ≥3 was considered (Guy et al., 2019; Zhang et al., 2020b). The incidence rate of anemia in the camrelizumab group was 3%, while the incidence rates of anemia, decreased neutrophil count, and vomiting in the chemotherapy group were 5.0, 15.0, and 5%, respectively (Huang et al., 2020). Other costs are shown in Table 1.

The utility value represents the health-related quality of life for each health state. The ESCORT trail did not involve health utility. Thus, the utility in the model was obtained from other public literature (Tan et al., 2018; Zhang et al., 2020b; National Institute for Health and Care Excellence, 2021), utility values for the PFD and PD health states were taken from EQ-5D data from a global, randomised, placebo-controlled, double-blind, phase 3 study, which recruited adults with advanced gastric cancer or gastro–oesophageal junction adenocarcinoma. The utility of PFD in the two groups was assumed to be consistent, but SAE (grade ≥3) could affect the utility. After disease progression, the utility of all patients in PD state was 0.581 (Zhang et al., 2020b; National Institute for Health and Care Excellence, 2021). All utility values are shown in Table 1.

### Sensitivity Analysis

One-way sensitivity analysis was performed to determine the influence of different parameters on ICER when changing within a certain range. The current price of camrelizumab fluctuated by 50% downward as the value range. The variation range of other parameters was the 95% confidence interval or the base value of the parameter ±25%. The discount rate was 0–8% (Liu, 2020). The results were presented in the form of tornado diagram. The horizontal axis of the cyclone graph represented the influence range of each parameter on ICER, and the vertical axis represented the parameter name. The degree of influence of the factors that have an influence on the evaluation result decreased from top to bottom.

In the probability sensitivity analysis, the parameters were set as random variables with specific distribution, and 10,000 Monte Carlo simulation was used to run the model to evaluate the overall robustness of the research results. The utility and the transition probability parameter were assumed to conform to the *β* distribution, and the cost parameter was assumed to conform to the *γ* distribution (Briggs et al., 2012). The results were represented by scatter plots and cost-acceptance curves.

## Results

### Basic-Case Analysis

Compared with the chemotherapy group, camrelizumab group showed an incremental cost of $1,439.64. The incremental effectiveness was 0.36 QALY and the ICER was $3,999.00/QALY in the first scenario. When both groups continued treatment until the disease progressed, the incremental cost of the camrelizumab group was $2,319.44 and the ICER was $6,442.89/QALY. In both scenarios, the ICER was far less than the WTP threshold of one time the GDP ($10503.52/QALY), that is, the camrelizumab group had an absolute cost-effective advantage. The results of basic-case analysis are shown in Table 2. The results of scenario analysis on a shorter time horizon are shown in Supplementary Table S2.

**TABLE 2:** Summary of base-case analyses.

Factor	Camrelizumab	Chemotherapy	Incremental camrelizumab vs. chemotherapy
QALYs			
Total	0.83	0.47	0.36
	0.59^a^	0.25^a^	0.34^a^
PFD	0.27	0.15	0.12
PD	0.56	0.32	0.24
LY	1.33	0.80	0.53
	0.92^a^	0.42^a^	0.50^a^
Costs (US, $)^b^			
Total	4,643.77	3,204.13	1,439.64
	3,872.10^a^	2,452.95^a^	1,419.15^a^
PFD	2,781.21	2,143.88	637.33
PD	1,862.56	1,060.25	802.31
Drug	2,520.67	1,638.97	881.7
Follow-up	792.50	508.23	284.27
Best supportive treatment	1,293.62	736.39	557.23
SAE	36.98	320.54	−283.56
Costs (US, $)^c^			
Total	5,971.02	3,651.58	2,319.44
	5,210.87^a^	2,852.34^a^	2,358.53^a^
PFD	4,108.46	2,591.32	1,517.14
PD	1862.56	1,060.25	802.31
Drug	3,833.05	2,039.42	1,793.63
Follow-up	788.11	476.90	311.21
Best supportive treatment	1,293.63	736.40	557.23
SAE	56.23	398.86	−342.63
ICER, $/QALY			3,999.00^b^
			4,173.97^b^ ^,^ ^a^
			6,442.89^c^
			6,936.85^c^ ^,^ ^a^

QALYs, quality-adjusted life-years; PFD, progression-free disease; PD, progressive disease; LY, life years; SAE, severe adverse event; ICER, incremental cost-effectiveness ratio.

a Results of the Weibull survival model; Unlabeled: Results of the loglogistic survival model.

b First scenario: camrelizumab for six courses, docetaxel for three courses, and irinotecan for four courses.

c Second scenario: treatment until the disease progressed.

### Sensitivity Analysis

**FIGURE 2:**
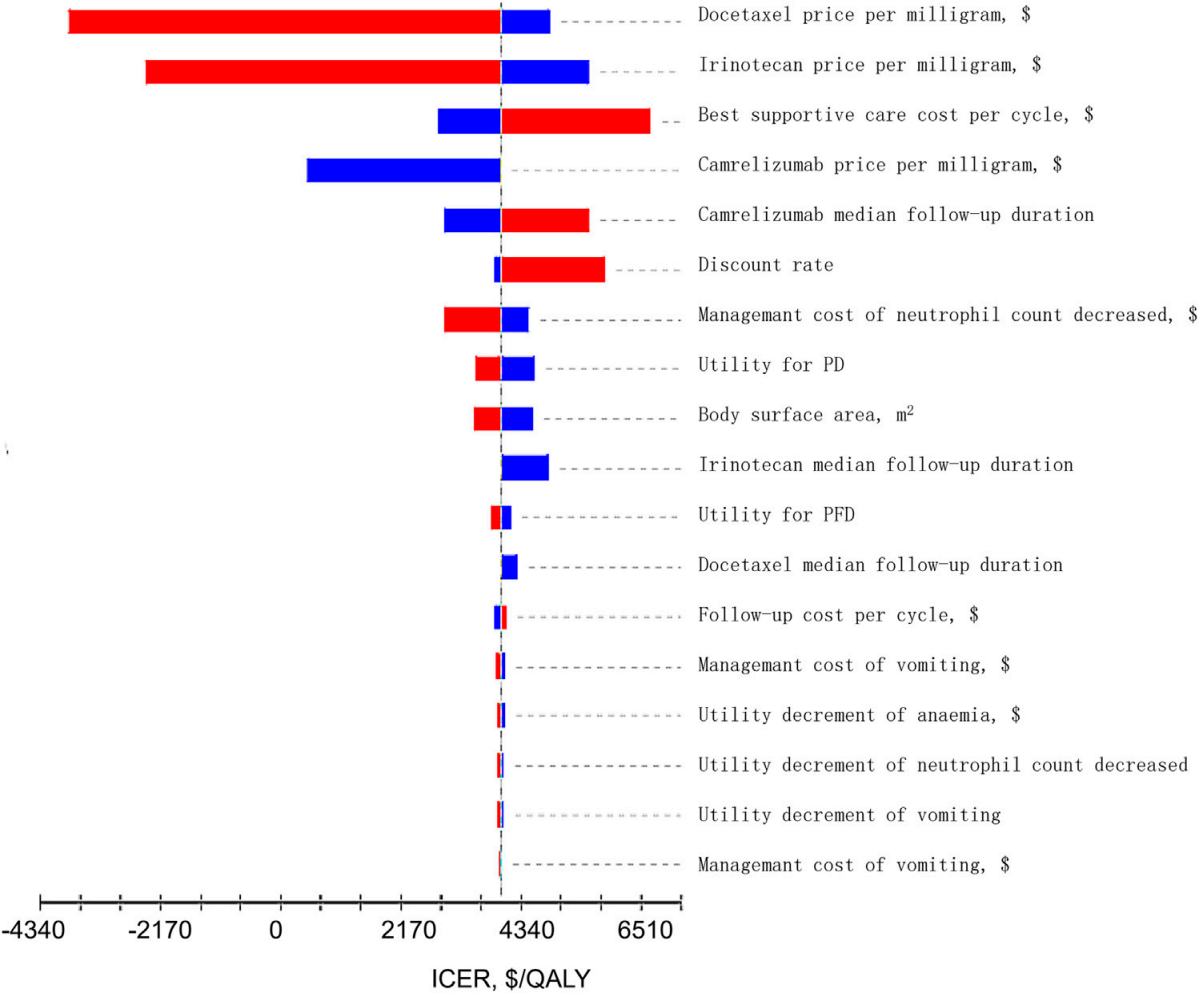
Tornado Diagrams of Univariable Sensitivity Analyses. The dotted line intersecting the red and blue bars represents the ICER of $3,999.00 per QALY from the base case results. The red bars represents the influence of the parameter on the result after the base value rises. The blue bars represents the influence of the parameter on the result after the base value drops.

The results of one-way sensitivity analysis are shown in Figure 2. The main factors with a great effect on ICER were the cost of docetaxel, irinotecan, best supportive treatment, and camrelizumab. The ICER value of the model did not exceed the threshold of one time per capita GDP with the change in all uncertainty parameters, basically consistent with the conclusion of basic-case analysis.

**FIGURE 3:**
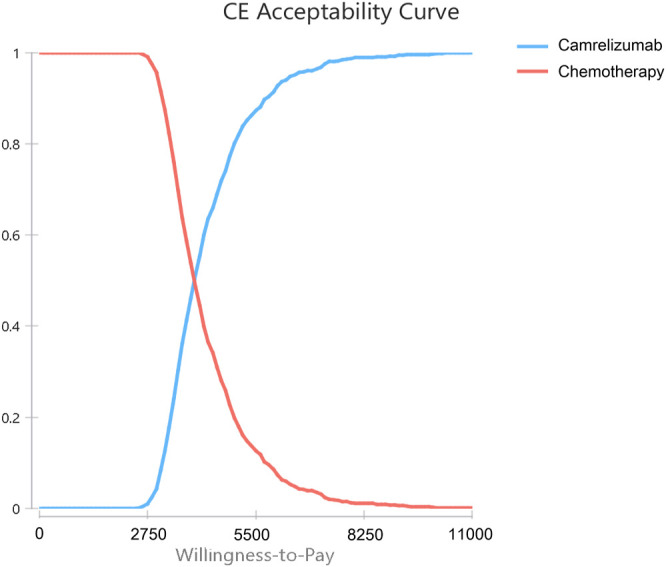
Cost-effectiveness Acceptability Curves. Results of probabilistic sensitivity analyses for camrelizumab versus chemotherapy after 10,000 Monte Carlo simulations, which indicates the probability of cost-effectiveness at different willingness-to-pay thresholds based on the uncertainty of the parameters.

The results of probabilistic sensitivity analysis are shown in Supplementary Figure S3. All the scatter points in the scatter plot were below the threshold, Supplementary Figure S3 indicating that camrelizumab was more cost-effective than chemotherapy in all cases. The cost-effectiveness acceptance curve is showed in Figure 3. When the WTP threshold was $3,151.06, the probability of cost-effectiveness advantage of camrelizumab was 14.4%. When the WTP threshold was $6,302.11, the probability of cost-effectiveness advantage was 92.5%. With the increase in the threshold, the possibility of camrelizumab to cost-effective increased; when the WTP threshold was $10,503.52, the probability of cost-effectiveness advantage was 100%.

## Discussion

Tumor immunotherapy, especially PD-1/PD-L1 ICIs, has shown rapid progress in the field of tumor treatment in recent years, and it has become another important tumor treatment method after surgery, radiotherapy, and chemotherapy. Considering the expected growth of immunotherapy for cancer treatment in the next few years, expenditures on patients with cancer could undoubtedly increase. The high cost of ICI treatment also brings a heavy financial burden to patients and their families. Some patients are forced to abandon ICI treatment because they could not afford it. Anticancer drugs should be more reasonable, inexpensive, and beneficial to people to finally realize their therapeutic significance. Camrelizumab entered China’s National Medical Insurance catalog for the first time, and it was the only ICI to enter this catalog. The original price of camrelizumab was $2,870.52 per 200 mg, and after price reduction, it was $424.51 per 200 mg (Nation Healthcare Security Administration, 2020). The price reduction is very large. It reduces the economic burden of patient medication, thus considerably increasing the availability of ICIs. Compared with traditional chemotherapy, camrelizumab could effectively prolong the OS and improve the objective remission rate. Therefore, evaluating its cost efficiency is necessary. In the present study, the cost efficiency of camrelizumab in the treatment of ESCC was evaluated for the first time through the establishment of an economic model method and the synthesis of the latest evidence.

The optimal treatment cycle of ICIs is currently unclear. Patients who discontinue ICIs due to toxicity or other reasons may continue to show clinical benefit (Emens et al., 2017). In the established model, the influence of the duration of drug treatment on the results was considered. The results showed that compared with the ICER value of chemotherapeutic regimens, that of camrelizumab was lower than the WTP threshold of one time the GDP per capita, and camrelizumab demonstrated an absolute cost-effectiveness advantage in all scenarios. The price of drugs was the most important influencing factor, but the various parameters in the model did not have a major effect on the results of the model.

In accordance with the recommendations of the 2020 edition of the Chinese Pharmacoeconomics Guide (Liu, 2020), the present study adopted per capita GDP as the threshold of WTP. On the basis of China’s national conditions, whether a higher threshold of WTP should be adopted as the evaluation standard for cancer drugs remains to be further explored. The cost-acceptance curve indicated that the possibility of cost efficiency of camrelizumab could be further improved with the increase in threshold, and the conclusion of the model did not change.

At present, the economic research on ICIs for the treatment of esophageal cancer is very limited. In a recent study, a Markov model was established to compare the cost effectiveness of nivolumab and chemotherapy from the perspective of Chinese society. Compared with chemotherapy, nivolumab increased by 0.107 QALYs and US$14,627.90, and the ICER was US$136,709.35/QALY. With a threshold of US$29,306.43/QALY, nivolumab may not have the cost-effective advantage (Zhang et al., 2020b). Compared with previous studies, the present study evaluated the cost efficiency of camrelizumab in the treatment of esophageal squamous cell carcinoma by using the partitioned survival model and combining with the best clinical evidence. The main reason for the difference between the results of nivolumab and that of the present study is that the ICIs of the two studies are different. Nivolumab is not included in the National Medical Insurance catalog, and the price difference between nivolumab and camrelizumab is very large. Differences in clinical efficacy and safety also exist between the two drugs, and the angle of study differs. These differences may be the reasons for the inconsistency between the two studies.

This study still has certain limitations. First, the model survival data originated from a published phase 3 clinical trial, and any bias in the trial could affect the results of this study. ESCORT is the only multicenter phase III clinical trial investigating camrelizumab in the treatment of esophageal squamous cell carcinoma. Given the strict inclusion and exclusion criteria, such as being younger and having fewer complications, patients entering clinical trials may be different from real-world patients. However, the ESCORT study is the best clinical evidence that could be found thus far, and it is a large-scale clinical study with good design. We did not have access to individual patient data from the ESCORT trials. Digitalization of the reported survival curves was used to replicate the survival data. This approach provides a reasonable, although not perfect, approximation to the actual survival data observed in the trials. The study explored alternative approaches to modelling survival such as scenario analyses using the loglogistic and Weibull distributions. The Weibull distribution gave similar results to the base-case analysis. Although there is a wide range of other functions available, these models performed reasonably well when compared with the observed survival. Therefore, the survival curve based on ESCORT research and simulation still has good accuracy and credibility. Second, no other head-to-head clinical trials were available. This study failed to compare the cost efficiency of camrelizumab with other treatment options. Third, different treatment options may be used after the disease progresses. The guidelines do not specify the third-line treatment plan for advanced or metastatic esophageal squamous cell carcinoma, and the third-line treatment plan is more complicated in clinical practice. The treatment plan after disease progression in the study was assumed to be the best supportive treatment to simplify the model, which may be different from the true state of the disease treatment. Fourth, only the SAE of grade 3 and above was considered when calculating cost and utility. The adverse events of grades 1 and 2 are usually mild, and they have a minimal effect on cost and utility. One-way sensitivity analysis showed that the results were not sensitive to the relevant parameters of SAE. Given that the results of this assessment reflected the general clinical practice of advanced esophageal squamous cell carcinoma, they may be valuable references for doctors and decision makers.

## Conclusion

Compared with chemotherapy, the second-line treatment of advanced or metastatic esophageal squamous cell carcinoma with camrelizumab not only could improve the quality of life of patients and prolong their survival time but also reduce the incidence of adverse reactions. It also has a cost-effective advantage in Chinese population.

## Data Availability

The original contributions presented in the study are included in the article/Supplementary Material, further inquiries can be directed to the corresponding author.
