# Supplementary material for: Cost-Effectiveness Analysis of Camrelizumab Versus Chemotherapy as Second-Line Treatment of Advanced or Metastatic Esophageal Squamous Cell Carcinoma
Source: Front Pharmacol. 2021 Nov 16;12:732912. doi: 10.3389/fphar.2021.732912 (PMC8634950; doi:10.3389/fphar.2021.732912)
Supplement: Supplementary file 1 [file Table1.DOCX]

**Supplementary Table A.1. Comparison of survival models**

|  | AIC | | BIC | |
| --- | --- | --- | --- | --- |
|  | Camrelizumab | Chemotherapy | Camrelizumab | Chemotherapy |
| OS |  |  |  |  |
| Exponential | 1302.478 | 1335.24 | 1305.902 | 1338.643 |
| Weibull | 1288.783 | 1304.384 | 1292.208 | 1307.787 |
| Logistic | 1375.81 | 1382.023 | 1379.235 | 1385.426 |
| Log-normal | 1282.201 | 1289.716 | 1285.626 | 1293.119 |
| Log-logistic | 1281.149 | 1287.884 | 1284.574 | 1291.286 |
| PFS |  |  |  |  |
| Exponential | 1116.008 | 858.4323 | 1119.433 | 861.7983 |
| Weibull | 1113.994 | 817.2631 | 1117.419 | 820.629 |
| Logistic | 1273.853 | 927.5635 | 1277.278 | 930.9294 |
| Log-normal | 1064.17 | 749.1523 | 1067.595 | 752.5182 |
| Log-logistic | 1061.409 | 738.6277 | 1064.834 | 741.9936 |

AIC: Akaike information criterion; BIC: Bayesian Information Criterion; OS: Overall survival; PFS: Progression-free survival;
