# Supplementary figures and images for: Cost-Effectiveness Analysis of Camrelizumab Versus Chemotherapy as Second-Line Treatment of Advanced or Metastatic Esophageal Squamous Cell Carcinoma
Source: Front Pharmacol. 2021 Nov 16;12:732912. doi: 10.3389/fphar.2021.732912 (PMC8634950; doi:10.3389/fphar.2021.732912)

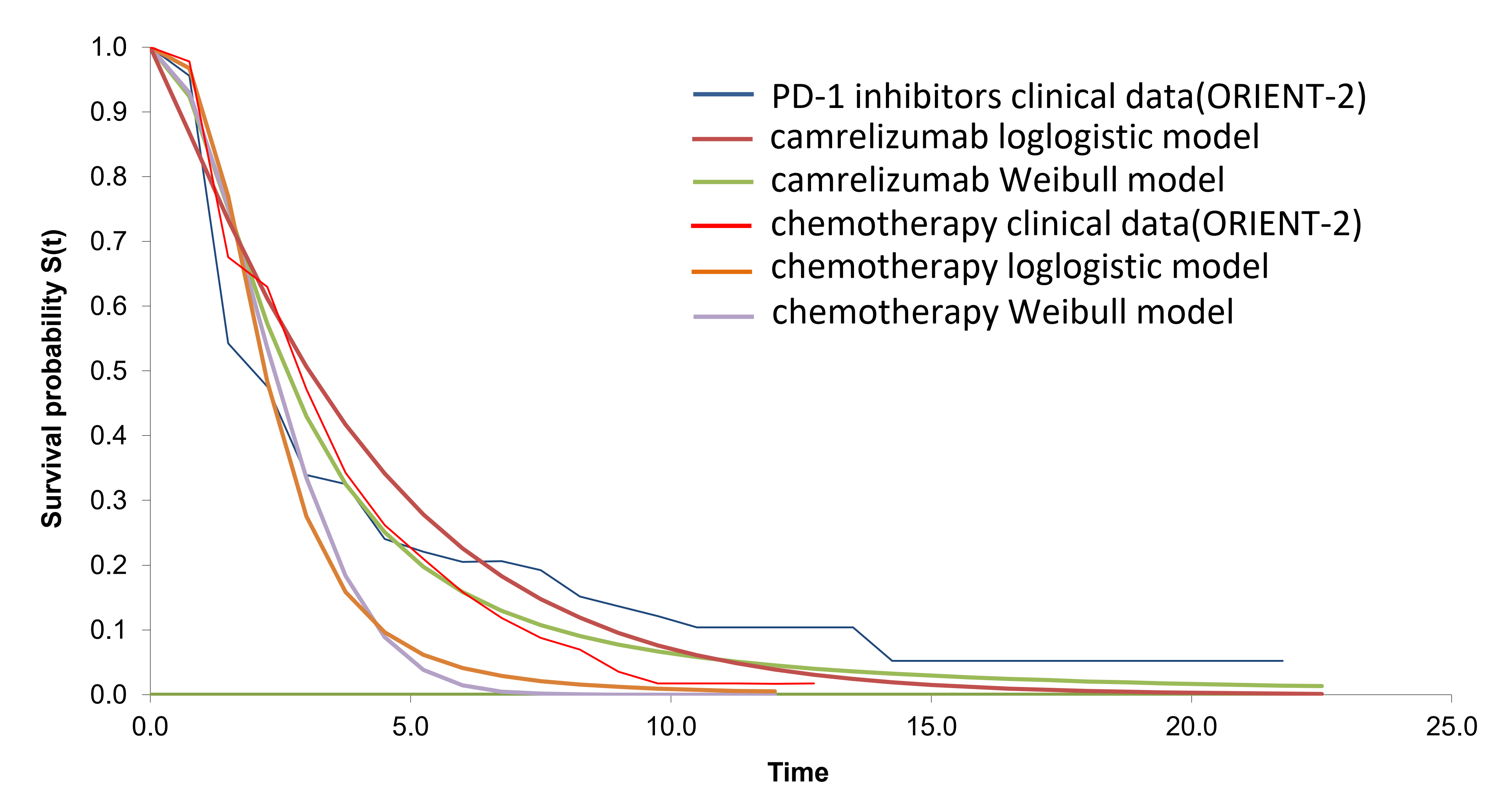

Supplement: Supplementary file 2 [file Image3.TIF]

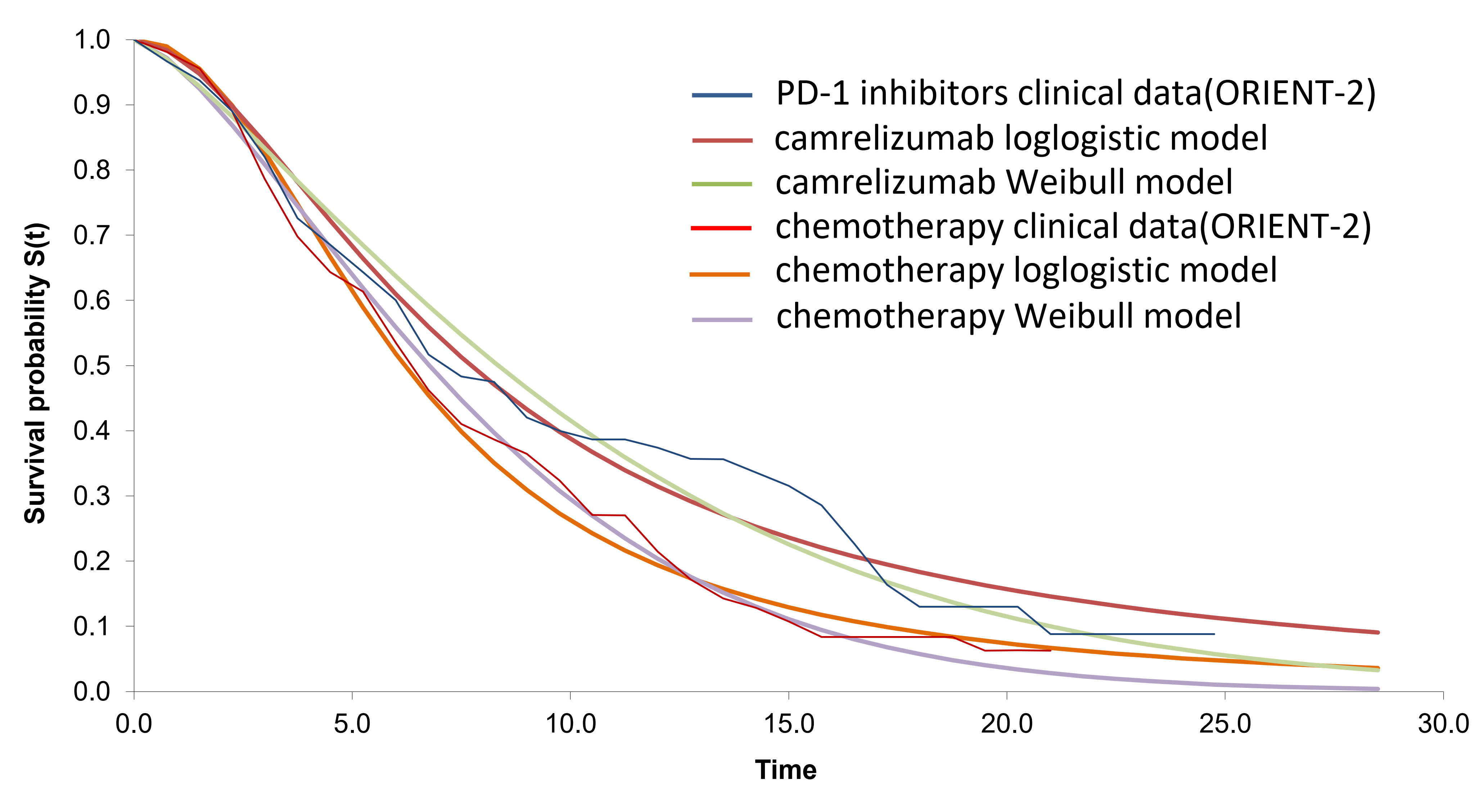

Supplement: Supplementary file 3 [file Image4.TIF]

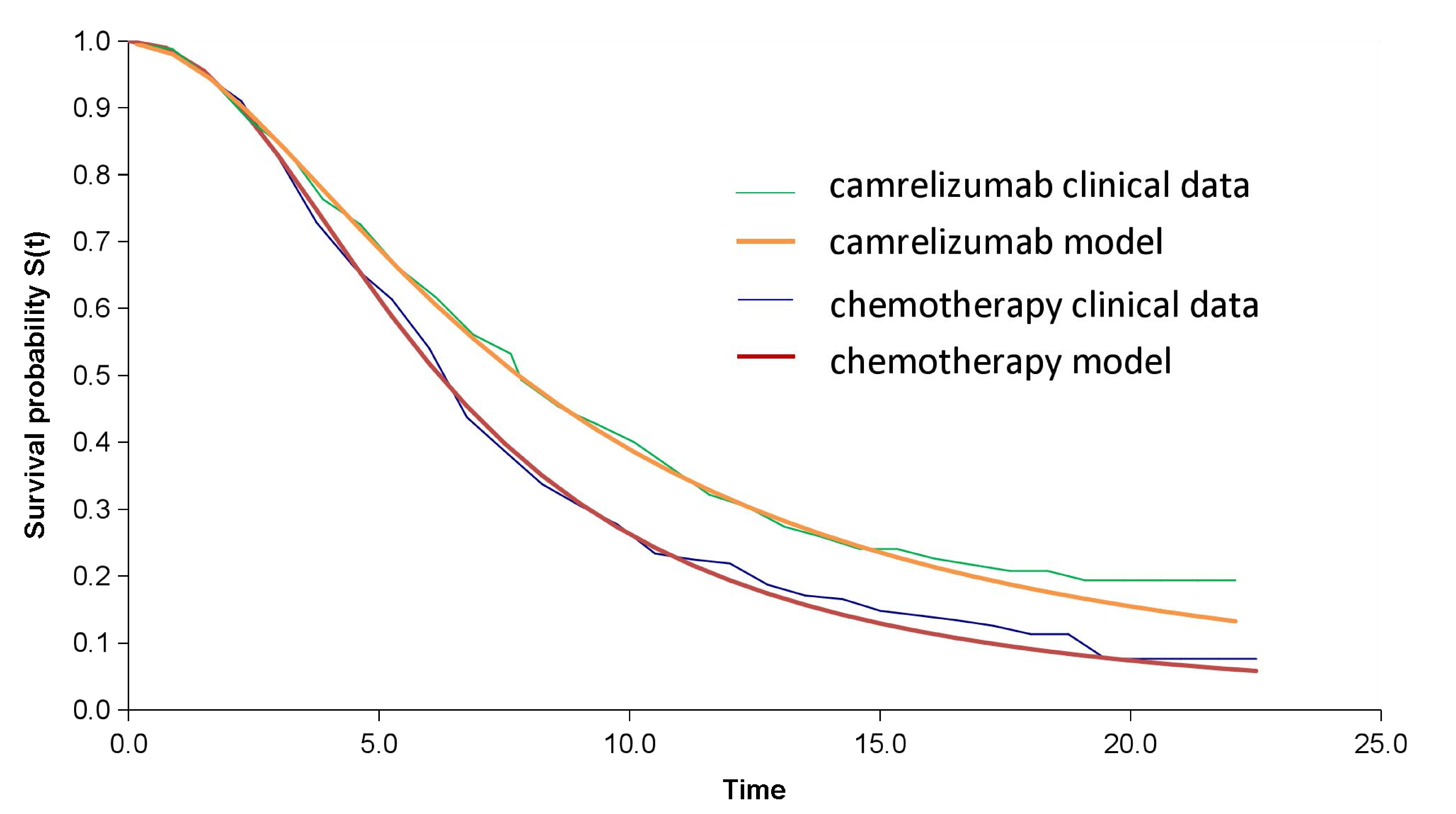

Supplement: Supplementary file 4 [file Image2.TIF]

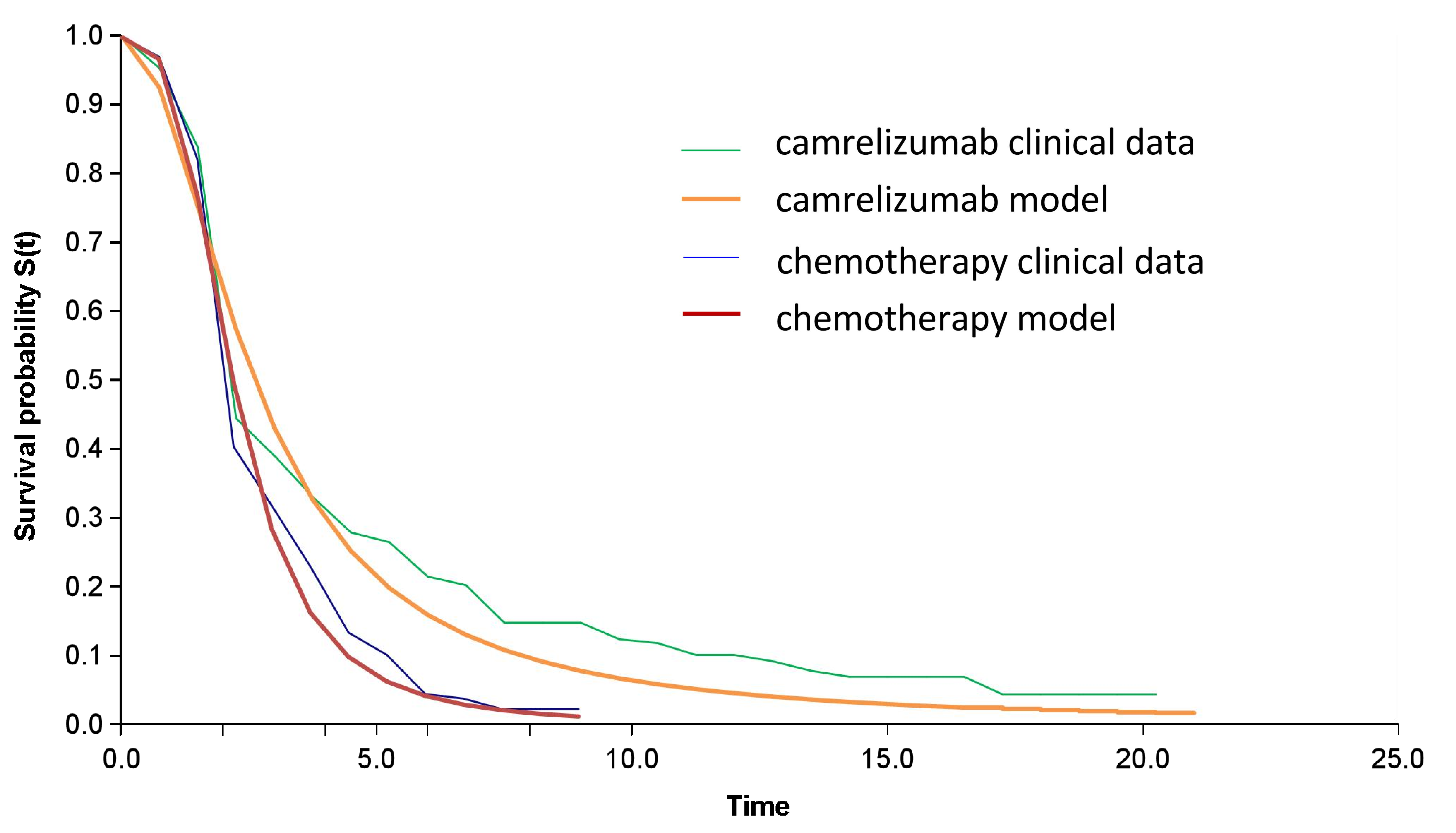

Supplement: Supplementary file 5 [file Image1.TIF]

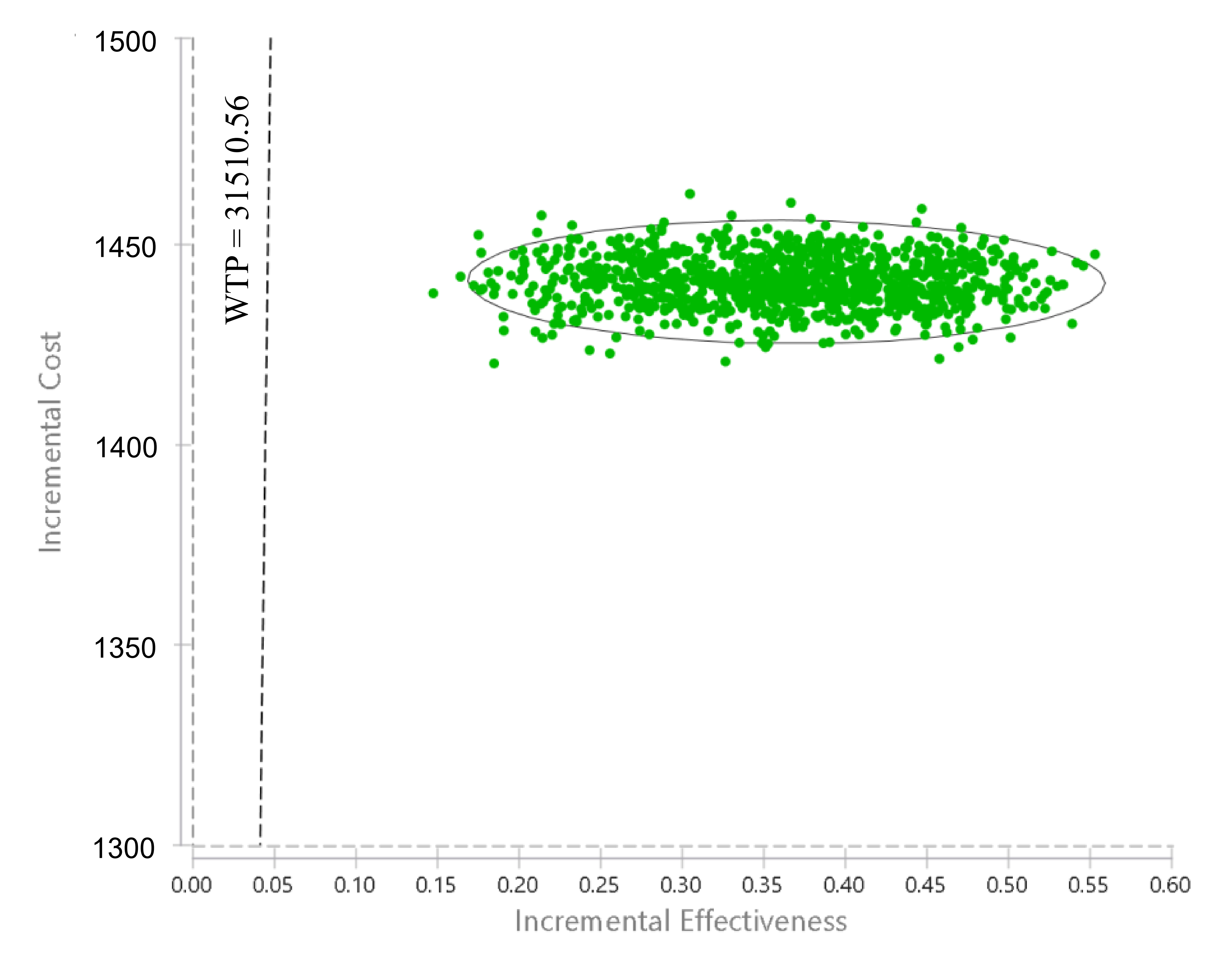

Supplement: Supplementary file 7 [file Image5.TIF]
