# Supplementary material for: Cost-Effectiveness Analysis of Camrelizumab Versus Chemotherapy as Second-Line Treatment of Advanced or Metastatic Esophageal Squamous Cell Carcinoma
Source: Front Pharmacol. 2021 Nov 16;12:732912. doi: 10.3389/fphar.2021.732912 (PMC8634950; doi:10.3389/fphar.2021.732912)
Supplement: Supplementary file 6 [file Table2.DOCX]

**Supplementary Table A.2. Scenario Analysis on a Shorter Time Horizon**

| Time horizon | Factor | Camrelizumab | Chemotherapy | Incremental  camrelizumab vs chemotherapy |
| --- | --- | --- | --- | --- |
| 3 years | QALYs | 0.64 | 0.39 | 0.25 |
|  | LY | 1 | 0.68 | 0.33 |
|  | Costs (US, $) | 5271.17 | 3400.15 | 1871.02 |
|  | ICER, $/QALY |  |  | 7484.09 |
| 5 years | QALYs | 0.72 | 0.43 | 0.29 |
|  | LY | 1.15 | 0.73 | 0.41 |
|  | Costs (US, $) | 5588.50 | 3516.24 | 2072.25 |
|  | ICER, $/QALY |  |  | 7145.70 |
| 7 years | QALYs | 0.78 | 0.45 | 0.33 |
|  | LY | 1.24 | 0.77 | 0.47 |
|  | Costs (US, $) | 5781.94 | 3585.95 | 2195.99 |
|  | ICER, $/QALY |  |  | 6654.51 |

QALYs: quality-adjusted life-years; LY: life years; ICER: incremental cost-effectiveness ratio
